# A comparative study: tongue muscle performance in weightlifters and runners

**DOI:** 10.14814/phy2.13923

**Published:** 2018-11-20

**Authors:** Heidi A. VanRavenhorst‐Bell, Kathy L. Coufal, Jeremy A. Patterson, Antje S. Mefferd

**Affiliations:** ^1^ Department of Human Performance Studies Wichita State University Wichita KS; ^2^ Department of Special Education & Communication Disorders, Education University of Nebraska Omaha NE; ^3^ Department of Hearing and Speech Sciences Vanderbilt University Nashville TN

**Keywords:** Exercise mode, tongue endurance, tongue strength

## Abstract

Exercise mode (i.e., resistance training, endurance training) is known to yield mode‐specific effects on strength and endurance of muscles that are directly targeted during the exercise. Such mode‐specific effects can also be observed in indirectly involved (i.e., nontargeted) muscles. Mode‐specific muscle performance changes of nontargeted muscles, however, have only been investigated within the skeletal system. Therefore, as a first step, this study aimed to determine if bulbar muscle performance (tongue strength [TS], tongue endurance [TE]) differs between weightlifters and runners and if group differences are tongue region‐specific. The Iowa Oral Performance Instrument (IOPI) was used to measure TS and TE of the anterior and posterior tongue regions in 21 weightlifters and 23 runners. In weightlifters anterior TS was significantly greater than posterior TS (*P = *0.008), whereas in runners anterior and posterior TS were comparable. Furthermore, weightlifters produced significantly greater anterior TS than runners (*P* = 0.001). Finally, TE was overall significantly greater in runners than in weightlifters (*P *= 0.001). Findings suggest that exercise mode may differentially impact performance patterns of nontargeted bulbar muscles. More research is warranted to better understand the mechanisms underlying tongue muscle performance differences between weightlifters and runners.

## Introduction

Maintaining healthy tongue muscle performance (e.g., tongue strength [TS], tongue endurance [TE]) is important for daily functional tasks such as swallowing and upper airway patency (Blumen et al. 2004; Munn et al. 2005; Stierwalt and Youmans 2007). TS and endurance, however, decline as a natural part of the overall aging process (Crow and Ship 1996; Clark and Solomon 2012). In the presence of significant decline, the risk of functional disorders such as dysphagia and sleep apnea, is greatly increased (Blumen et al. 2004; Munn et al. 2005; Clark and Solomon 2012; McSharry et al. 2012).

In the skeletal system (e.g., limb and trunk muscles), exercise (i.e., resistance training, running) is known to have a *direct and indirect* beneficial effect on muscular strength and endurance, in addition to retarding the aging process (Takeshima et al. 2007; Tew et al. 2009; Karavirta et al. 2011b). Recently, exercise has been shown to have a similar *indirect* beneficial effect on tongue muscle strength and endurance in rats (Kletzien et al. 2013). Furthermore, in humans, VanRavenhorst‐Bell et al. (2017) reported that tongue muscle performance measures were significantly higher in individuals who reportedly engaged in high levels of physical activity compared to individuals who reported engaging in low levels of physical activity. Most importantly, this study found that the difference in tongue performance of individuals with high and low physical activity levels was more pronounced in older adults than in younger adults.

When examining the effect of exercise on muscle performance, however, several factors such as *mode, intensity, duration,* and *frequency* have to be considered. These factors are known to modulate the effect of exercise on *directly involved (targeted)* as well as *indirectly involved (nontargeted)* skeletal muscles, including exercise‐related changes in strength and endurance (Tew et al. 2009; Karavirta et al. 2011a; Wilson et al. 2012b). Although the effect of some of these factors has been examined when the exercise was designed to directly target tongue muscles (Clark 2012; Van Nuffelen et al. 2015; Slovarp et al. 2016), it is currently unknown if these factors also impact tongue muscle performance when exercise targets skeletal muscles and only indirectly involves tongue muscles. Such information would be valuable to better understand how to promote healthy tongue muscle performance throughout the aging process. In addition, it could potentially be used to compliment current rehabilitative methods aimed at improving tongue muscle strength and endurance when loss has occurred.

### Differential effects of exercise mode on muscle strength and endurance

In the skeletal system (e.g., limb and trunk muscles), exercise is known to beneficially affect muscular strength and endurance and retard the aging process (Takeshima et al. 2007; Karavirta et al. 2011b). Exercise is capable of benefiting muscles that are *directly* targeted, as well as muscles that are *indirectly* involved during exercise (Munn et al. 2004; Tew et al. 2009). For example, performing unilateral arm flexor curls, a form of resistance training, directly *targeting* the arm muscle strength of one arm was further shown to *indirectly* benefit the muscular strength of the elbow flexors of the nonexercised armed (Munn et al. 2005; Carroll et al. 2006).

The effect of exercise on skeletal muscle strength and endurance, however, can depend on several factors (e.g., *mode, intensity, duration, frequency*). Although each exercise factor elicits muscle fiber adaptations that affect muscle strength and muscle endurance differently (Karavirta et al. 2011a), the current study will only focus on exercise mode; specifically, resistance training (weightlifting) and endurance training (distance running).

Previous studies have demonstrated that resistance training (i.e., weightlifting) has a greater effect on Type II fast‐twitch muscle fibers and therefore improves muscular strength, speed, and power; whereas endurance training (i.e., long‐distance running) has a greater effect on Type I slow‐twitch muscle fibers resulting in more efficient utilization of oxygen and a lower rate of fatigue (Holloszy and Coyle 1984; Wilson et al. 2012a).

#### Resistance training

Resistance training is a mode of physical exercise that engages one or more muscle groups to contract against an external force followed by a brief period of rest. A form of resistance training known as isotonic resistance training involves shortening (concentric) and lengthening (eccentric) of a muscle(s) while it contracts against an external force throughout the entire range of motion. Due to the resistive load being applied to the muscle(s) during each contraction the exercise challenges the muscle, which results in hypertrophy of muscle fibers and hyperplasia (increased cell production).

Resistance exercise, however, not only impacts the muscles that are *directly* targeted but also those that are *indirectly* involved. For example, *direct* resistance training on one limb increased muscle strength in the exercised limb as well as small yet robust increases in muscle strength in the contralateral *nonexercised* limb (Munn et al. 2004, 2005; Carroll et al. 2006). Such findings have been shown to occur in upper extremity (i.e., elbow flexor) and lower extremity (i.e., knee extensors) alike (Munn et al. 2005; Carroll et al. 2006).

#### Endurance training

Endurance training is a mode of exercise that involves repetitive contractions of a muscle or group of muscles over an extended duration of time. Each contraction is executed at a level of effort that is below that of maximal capacity (i.e., submaximal contraction). The prolonged submaximal muscle contractions result in more efficient utilization of oxygen by muscle cells, which delay the onset of muscle fatigue. In turn, an individual can perform muscle contractions for a longer duration of time and at a greater intensity.

Numerous studies have shown that the muscles responsible for locomotion have a greater number of slow‐twitch type I fibers and produce a greater volume of oxygen consumed (VO_2_) in endurance‐trained individuals (i.e., distance runners) than resistance‐trained and untrained individuals (Demirel et al. 1999; Wilson et al. 2012a,b). Additionally, endurance exercise elicits muscle fiber adaptations driven by the need for more efficient use of oxygen in the *directly* targeted muscle(s) as well as the muscle fibers in *nontargeted* muscle(s) (Holloszy and Coyle 1984; Demirel et al. 1999; Wilson et al. 2012a). The adaptive muscle fibers contribute to an increase in muscle endurance in *targeted* and *nontargeted* muscles.

### Anatomical fiber composition and functional performance of tongue musculature

The tongue is a complex structure that is commonly conceptualized as consisting of two distinct regions (Stal et al. 2003). The anterior tongue region (anterior two‐third) is comprised predominantly of Type II fast‐twitch muscle fibers (Stal et al. 2003). These fibers have been shown to be proficient in performing movements purposed toward strength, power, and speed (Stal et al. 2003; Zaidi et al. 2013). Therefore, these fibers are predominantly found in the anterior tongue region where they can accommodate functions such as bolus manipulation and propulsion during swallowing as well as articulatory movements during speech production.

In contrast to the anterior region of the tongue, the posterior region is comprised primarily of Type I and Type IM/IC slow‐twitch muscle fibers (Stal et al. 2003). These slow‐twitch muscle fibers are known for being relatively fatigue resistant and are therefore well suited for functions carried out by the posterior region of the tongue, such as maintaining an open upper airway during respiration and closing off the upper airway during mastication to prevent premature advancement of the bolus (Stal et al. 2003; Zaidi et al. 2013).

#### Tongue muscle strength

TS is a measure that relates to the amount of force the tongue muscle fibers produce (Stierwalt and Youmans 2007; Clark 2012; Adams et al. 2013). TS within the anterior region has been shown to be slightly higher than that of the posterior region, yet TS values for both regions are collectively reported (Clark and Solomon 2012; Adams et al. 2013, 2014). Maximal TS values for both the anterior and posterior regions are shown to vary between 40–80 kPa in healthy adults (18–59 years of age) with 60 kPa representing the average maximal TS (Crow and Ship 1996; Clark and Solomon 2012; Adams et al. 2013). These values, however, are shown to decline gradually, an average of 10–15 kPa, with increasing age in healthy adults 60 years of age and older (Crow and Ship 1996; Clark and Solomon 2012; Adams et al. 2013). Furthermore, a TS value declining below 35 kPa is viewed as a concern for producing adequate tongue function, such as swallowing (Adams et al. 2013).

#### Tongue muscle endurance

TE is defined as the length of time one can maintain a submaximal contraction (Crow and Ship 1996; Stierwalt and Youmans 2007; Clark 2012). From the initial moment the muscle contracts, the process of fatigue begins and may compromise lingual function if tongue muscle endurance is not adequate (Bigland‐Ritchie et al. 1986). Although only a few studies have addressed TE and a widely accepted consensus is still lacking, some experts suggested that healthy individuals, regardless of age, should be able to maintain approximately 50% of their maximum TS for at least 25–35 sec when measured either at the anterior or posterior regions (Crow and Ship 1996; Stierwalt and Youmans 2007; Adams et al. 2013). A few studies have shown that the anterior region is able to maintain a slightly longer TE measure than the posterior region (Adams et al. 2013, 2014). Regardless of regional TE differences, a performance of 10 sec or less on such a TE test is viewed as a concern for maintenance of adequate tongue function (Crow and Ship 1996; Stierwalt and Youmans 2007; Adams et al. 2013).

### Mode of exercise and tongue muscle performance

It is known that tongue muscles are *indirectly* involved in physical exercise. For example, running not only increases skeletal muscle activity in the arms and legs; it also results in increased tongue muscle activation to dilate the upper airway for more efficient gas exchange (Shi et al. 1998; Williams et al. 2000). The effect of exercise on tongue muscle performance has been studied recently by Kletzien et al. (2013). They reported beneficial effects of exercise on TS and TE of rats following *direct* isotonic resistance training as well as *indirect* endurance training (treadmill running; Kletzien et al. 2013). Furthermore, VanRavenhorst‐Bell et al. (2017) reported that individuals (humans) who regularly engaged in physical activity had tongue muscle performance measures that were greater than those that did not engage in physical activity, thereby, supporting that physical exercise may have a potential indirect effect on tongue muscle performance. To continue to better understand the potential indirect effect of physical exercise on tongue muscle performance it is important to look further into exercise mode as a factor differentially impacting TS and endurance.

#### Tongue muscle involvement during physical activity

Tongue muscles are activated during breathing to regulate airway patency (Shi et al. 1998; Williams et al. 2000). Depending on the mode of exercise, breathing patterns and requirements for airway patency vary (Ikeda et al. 2009; Kalsas and Thorsen 2009; Elliott and Grace 2010). For example, weightlifting (isotonic resistance training) is associated with an intense and forceful breathing pattern often referred to as the Valsalva maneuver. This breathing pattern includes several phases, beginning with a forceful inhalation followed by a brief pause during which the glottis may be tightly sealed to prevent air leakage, and then a forceful exhalation through an open glottis (Ikeda et al. 2009; Kawabata et al. 2010; Talasz et al. 2011). Hence, presumably forceful breathing (i.e., Valsalva maneuver) requires rapid adjustments of the airway during forceful inhalation and exhalation as well as brief, relatively high supralaryngeal muscle forces assisting with the glottal closure to withstand the high subglottal air pressures.

In contrast, endurance runners engage in a rhythmic pattern of breathing known as *coupling* (Bernasconi and Kohl 1993; Elliott and Grace 2010; Daley et al. 2013). Specifically, runners synchronize their leg movements with the respiratory frequency in a phase‐locked pattern; for example, four strides per breath, three strides per breath, or two strides per breath (Daley et al. 2013). Such coupling requires tongue muscles to contract at a submaximal level of effort over an extended period of time with minimal fatigue (Holloszy and Coyle 1984; Jones and Carter 2000). Acknowledging that the demands on the tongue musculature differ between the Valsalva maneuver during weightlifting and the coupled breathing during running and that these demands may elicit mode‐specific adaptive responses in the muscle fibers of the tongue, different performance patterns of TS and endurance may be observable between weightlifters and runners.

### Purpose statement

The purpose of this study was to begin examining exercise mode as one factor that can impact muscle performance patterns of nontargeted bulbar muscles. As a starting point, this study sought to determine if tongue muscle performance (i.e., strength, endurance) differs between individuals who regularly engage in resistance training exercise (e.g., weightlifting) and individuals who regularly engage in endurance training exercise (e.g., running). In addition, the study sought to determine if performance (i.e., strength, endurance) differs between anterior and posterior tongue musculature given the differences in muscle fiber composition in these tongue regions. Finally, it was of interest to examine if the tongue muscle performance pattern of a specific tongue region depended on group membership.

It was hypothesized that resistance‐trained weightlifters would produce greater TS than endurance‐trained runners, particularly in the anterior region of the tongue due to the specific muscle fiber distribution in this region. By contrast, endurance‐trained runners were expected to demonstrate greater TE compared to weightlifters, with the group difference being primarily observable in the posterior region of the tongue.

## Method

### Participants

Forty‐five healthy, male and female young adults (19–29 years of age) volunteered to participate and recruited through print materials and technology‐based communication. Facilities and organizations targeting the population known to engage in weightlifting (resistance mode of training) or long‐distance running (endurance mode of training) in the surrounding region were included. The Wichita State University Institutional Review Board approved the study and an informed consent form was obtained from all volunteers prior to completing any questionnaire(s) or participating in data collection.

*Inclusion and exclusion criteria*. The mode of exercise each volunteer regularly engaged in was identified based on the *Physical Activity Questionnaire* (MeDesign, 2013). To control for selection bias, the examiner followed a script. The volunteers were blind to the desired criterion necessary to participate in the study. Only volunteers who reported that they regularly engaged in resistance training (weightlifting) or endurance training (distance‐running) 4 or more days per week over the past year were included in this study. Individuals reporting a cross‐training exercise schedule (e.g., equal days of resistance training and endurance training, 3‐day/4‐day split of resistance training to endurance training) and individuals who reported training regularly for less than 1 year were not eligible for this study.

The *Physical Activity Readiness Questionnaire* (Canadian Society for Exercise Physiology, 2002) was used to identify and exclude volunteers with physical health concerns that could interfere with successfully completing the objective cardiorespiratory endurance assessment and/or volunteers taking medications that may alter the cardiorespiratory response during the objective assessment (e.g., diuretic, beta‐blocker). In addition to these health concerns and medications, volunteers were excluded if they reported a history of a neurological disorder or other disease(s) or injury(s) known to negatively impact lingual function.

All 45 volunteers passed the intake questionnaires and were included in this study. One male weightlifter, however, was unable to perform the necessary tasks for tongue measures and therefore, was removed from the study. Table 1 provides the demographics of the remaining participants in each group.

**Table 1:** Descriptive group means, standard deviations, and sum of weightlifters, runners and overall

Group	Gender	Age ± SD	*N*
Weightlifters	Male	24.09 ± 3.02	11
	Female	22.70 ± 3.23	10
	Total	23.43 ± 3.12	21
Runners	Male	23.92 ± 3.12	12
	Female	23.45 ± 3.01	11
	Total	23.70 ± 3.01	23
Overall	Male	24.00 ± 3.00	23
	Female	23.10 ± 3.06	21
	Total	23.57 ± 3.03	44

### Study design

To verify physiological performance differences between the two groups, measures of handgrip strength and cardiorespiratory endurance were obtained for each participant. To assess maximal handgrip strength a standardized protocol using a hand‐held dynamometer was administered (Ehrman 2010). To assess cardiorespiratory endurance the submaximal VO_2_ *Young Men's Christian Association* (YMCA) protocol was administered using a bicycle ergometer. The examiner (first author) who was trained and experienced in administering both of these protocols provided all instructions and collected the data for these measures.

The *Iowa Oral Performance Instrument* (IOPI) was used to assess anterior and posterior TS and endurance. Furthermore, maximal TS was measured three times at the anterior tongue region and three times at the posterior tongue region. TE was measured once at the anterior tongue region and once at the posterior tongue region (Robbins et al. 2007; Adams et al. 2014).

### Instrumentation

The BASELINE hydraulic handheld dynamometer (Fabrication Enterprises Inc., Irvington, NY) was used to obtain maximal handgrip strength. The Monark Ergomedic 828E cycle ergometer (Monark, Vansbro, Sweden) along with the YMCA Cycle Ergometer protocol was used to obtain the cardiorespiratory endurance measure. In addition, a PolarWearLink Coded heart rate sensor system (Polar Electro, Lake Success, NY) was synchronized with the Monark 828E to provide continuous monitoring of the participant's heart rate. As recommended by the manufacturer calibration of the Monark Ergomedic 828E was conducted prior to each test session. Finally, the IOPI 2.1 (IOPI Medical, Carnation, WA) was used to obtain TS and endurance measures. The IOPI was calibrated monthly, as recommended by the manufacturer.

### Procedures

#### Handgrip strength

To objectively verify differences in skeletal muscular strength between weightlifters and runners, the BASELINE hydraulic handheld dynamometer was used. Maximal handgrip strength was measured based on a standardized protocol (Ehrman 2010), which is described in detail in VanRavenhorst‐Bell et al. (2017). Briefly, all participants used their nondominant hand to apply maximal isometric force by squeezing the dynamometer for 2–3 sec. A total of three trials was completed followed by a 30‐sec rest period between trials to control for fatigue. Each participant's highest value was recorded as maximal handgrip strength (Ehrman 2010).

**Figure 1:**
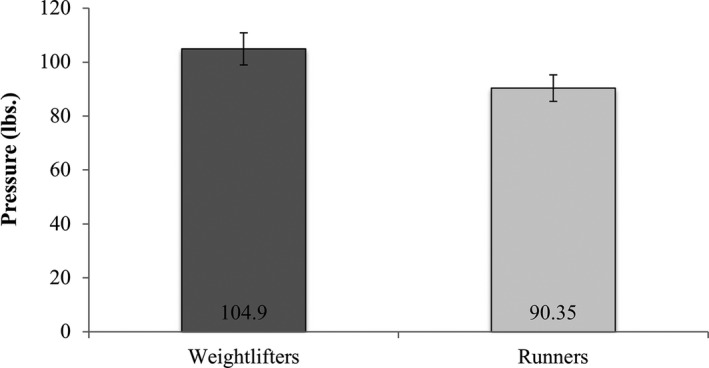
Means and standard errors of handgrip strength in weightlifters and runners.

To evaluate between‐group differences for handgrip strength, a two‐tailed independent *t*‐test was conducted. Statistical significance was approached [*t* (42) = 1.90, *P *= 0.07]. As can be seen in Figure 1 handgrip strength of the weightlifters (M* *=* *104.90 ± SE* *= 5.96) tended to be greater than handgrip strength of runners (M* *=* *90.35 ± SE* *= 4.92). Given the fact that the *P*‐value was based on a two‐tailed *t*‐test and the direction of the effect was in the expected direction, the findings for handgrip strength were interpreted to suggest distinct differences in skeletal muscles strength between these two groups.

#### Cardiovascular endurance

To objectively verify cardiorespiratory endurance differences between weightlifters and runners, the YMCA 85% submaximal cycle ergometer protocol was administered (see VanRavenhorst‐Bell et al. 2017 for more details). Briefly, during this standardized multistage test participants were asked to pedal on a MONARK cycle ergometer for 9–12 min at a steady pedaling cadence of 60 rotations per minute (rpm). The heart rate was monitored and dictated the workload for each 3‐min stage. The completion of at least two stages in addition to obtaining a steady state heart rate between 110 beats per minute (bpm) and 85% of age‐predicted maximum heart rate marked the end of the test (Ehrman 2010).

**Figure 2:**
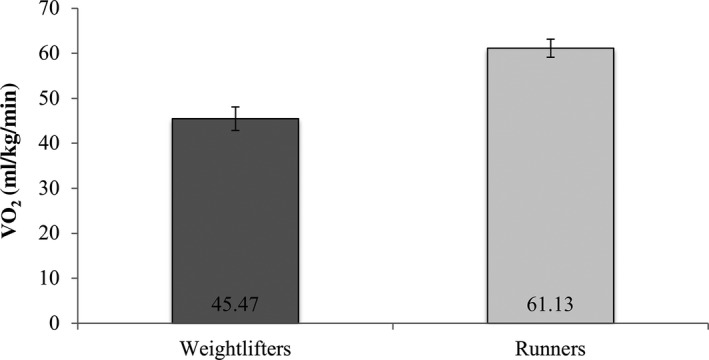
Means and standard errors of cardiovascular endurance in weightlifters and runners.

A two‐tailed independent *t‐*test was used to determine between‐group differences in cardiovascular endurance. As can be seen in Figure 2, a significant difference in cardiovascular endurance was found between weightlifters and runners [*t* (42)=−4.80, *P *< 0.001], with weightlifters producing a significantly lower estimated VO_2_ (M* *=* *45.47 ± SE* *= 5.96) than runners (M* *=* *61.13 ± SE* *= 4.92). This direction of effect for cardiovascular endurance supports the expected performance pattern for the two groups and indicates that these two groups indeed differed in cardiorespiratory endurance.

### Tongue muscle performance

After completion of the handgrip strength and cardiorespiratory endurance tasks, participants completed the TS and endurance measures. All tongue measures were obtained using the IOPI system and followed the same protocol that was described in detail in a previous study by VanRavenhorst‐Bell et al. (2017). Because it is known that the muscle fiber composition differs across the regions of the tongue (Robbins et al. 2007; Zaidi et al. 2013) maximal TS and TE was measured at the anterior location (10 mm posterior to the tongue tip) as well as the posterior location (10 mm anterior to the most posterior circumvallate papilla) (Robbins et al. 2007). The order of the tongue region was counterbalanced within each group.

#### Tongue strength

After proper placement of the bulb, participants were instructed to push the bulb against the roof of the mouth with their tongue and apply maximal static pressure for 1 or 2 sec. The examiner provided verbal cues during each trial, “push, push, push, good.” Three trials were completed for each tongue region. Between each trial, a 30‐sec rest was provided. The best of three measurement trials was recorded as the maximal TS (Robbins et al. 2007).

#### Tongue endurance

After the completion of TS measures participants were provided with a 1‐min resting period. Participant was instructed to maintain 50% of their maximal TS while pressing the IOPI bulb against the roof of the mouth for as long as possible. To assist with producing the desired tongue pressure against the bulb, participants were instructed to use the visual cues provided on the IOPI LED display. Sustaining a pressure that hovered around the desired measure was permissible, however, a pressure falling below the desired measure and failing to recover within 2 sec resulted in terminating the endurance trial. The duration during which TS was maintained at 50% maximal TS pressure was recorded as TE (Adams et al. 2014).

### Statistical analysis

Linear mixed effect models (LME) were run with group, tongue region, and group x tongue region as fixed effects. Separate models were run for each measure (TS, TE). Participants were added as random effects to account for multiple observations per participant. Specifically, because three trials were conducted to measure TS at each tongue region, a total of six values were submitted to the LME model for TS. However, only one trial was completed for TE at each tongue region; thus, participants were not included as a random effect in the LME model for TE. If interactions were found to be significant, paired comparisons were completed using additional LME models. The critical alpha level was set to *P ≤* 0.05 for all statistical tests.

## Result

### Tongue strength

**Figure 3:**
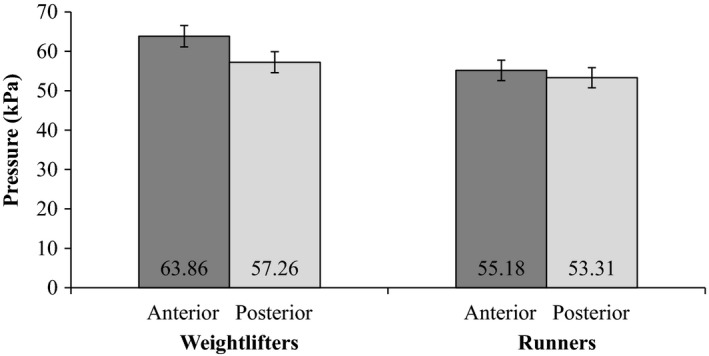
Means and standard errors of anterior and posterior tongue strength in weightlifters and runners.

A statistically significant main effect was found for tongue region on TS [*F* (1, 194.93) = 23.04, *P *< 0.001]. The main effect of group on TS was not statistically significant. The tongue region x group interaction, however, was significant [*F* (1194.93) = 7.19, *P *= 0.008]. As can be seen in Figure 3, in weightlifters the anterior TS was significantly greater than the posterior TS [Mean difference = 7.260, ±SE* *= 1.13; *F*(1, 68.39) = 41.01, *P *< 0.001], whereas in runners anterior and posterior TS were comparable. Furthermore, weightlifters had significantly greater TS in the anterior tongue region than runners [Mean difference = 8.95, ±SE* *= 3.05; *F*(1, 42.00) = 8.63, *P* = 0.005], whereas in the posterior tongue region TS was comparable across the two groups.

### Tongue endurance

**Figure 4:**
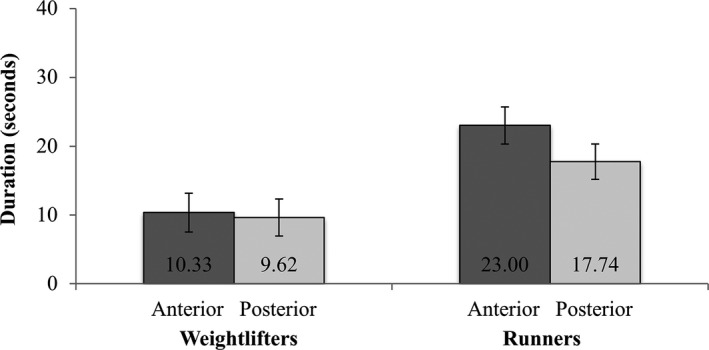
Means and standard errors of anterior and posterior tongue endurance in weightlifters and runners.

Identifying TE values were not normally distributed within each group, values were squareroot‐transformed prior to the statistical analysis. A statistically significant main effect of group on TE was found [*F* (1,83.323) = 19.83, *P *< 0.001]. As can be seen in Figure 4, the squareroot‐transformed TE of runners (M* *=* *4.24 ± SE* *= 0.22) was significantly greater than the squareroot‐transformed TE of weightlifters (M* *=* *2.80 ± SE* *= 0.23). The main effect of tongue region on TE and the group x tongue region interaction were not significant.

## Discussion

The purpose of this study was to determine (Adams et al. 2014) if tongue muscle performance differed between individuals who regularly engage in resistance training (e.g., weightlifting) and individuals who regularly engage in endurance training (e.g., distance running), (Adams et al. 2013) if tongue muscle performance differed between anterior and posterior tongue regions given the difference in muscle fiber composition, and (Bernasconi and Kohl 1993) if tongue muscle performance patterns of anterior and posterior tongue regions depended on group membership. Based on the review of literature it was expected that weightlifters would produce greater TS than runners, particularly in the anterior region of the tongue due to the specific muscle fiber distribution in this region. By contrast, runners would demonstrate greater TE compared to weightlifters, with the group difference being primarily observable in the posterior region of the tongue.

Hypotheses were supported in part for TS and TE measures. For TS, weightlifters demonstrated significantly greater TS than runners in the anterior region of the tongue, whereas between‐group differences in TS were comparable for the posterior tongue region. Furthermore, for TE a significant group effect was found with runners demonstrating significantly greater TE than weightlifters; however, not as expected predominantly in the posterior tongue region. Rather, TE tended to be greater in the anterior tongue region than posterior tongue region in runners; by contrast, in weightlifters anterior and posterior TE was rather comparable. These findings will be addressed in the following sections.

### Tongue strength

Findings for TS concurred with previous literature on skeletal musculature that reported muscle strength of *directly* and *indirectly* targeted muscles to be greater in resistance‐trained individuals (e.g., weightlifters) than endurance‐trained individuals (e.g., runners) (Munn et al. 2004; Karavirta et al. 2011a; Wilson et al. 2012a,b). It is important to point out, however, that both weightlifters and runners produced TS measures that fell well within the normative range of 40–80 kPa (Crow and Ship 1996; Stierwalt and Youmans 2007; Clark and Solomon 2012; Adams et al. 2013). If weightlifting engages the tongue sufficiently to produce a mode‐specific exercise effect, then perhaps values toward the upper limit of the normative range should be expected. Yet, it is also possible that the effect of weightlifting on TS is relatively subtle and extreme TS values may not be reasonable to expect. Other factors, such as the size of orofacial structures, may be a much more plausible explanation for extremely high TS values. To shed light on these questions, future studies are warranted that investigate the effect of resistance training (weightlifting) on TS directly; for example, by use of a pre‐ and postexercise design.

### Tongue endurance

Study outcomes revealed that runners produced significantly greater TE than weightlifters. This finding suggests that tongue musculature may respond to the *indirect* effects of exercise mode similar to that of skeletal musculature. Running has been shown to improve muscle endurance of *indirectly* targeted skeletal muscles (Jones and Carter 2000; Tew et al. 2009; Karavirta et al. 2011a,b), in part due to skeletal muscles contracting at a submaximal intensity for a longer duration of time compared to that of resistance training (weightlifting) (Karavirta et al. 2011; Wilson et al. 2012a,b). In addition, our finding parallels that of a prior study on rats, which showed that endurance training (running on treadmill) was associated with a significant increase in TE (Kletzien et al. 2013). Acknowledging that there are important differences in the head and neck anatomy and physiology between rats and humans the findings of the current study and those reported by Kletzien and colleagues, however, suggest that exercise may have an *indirect* effect on tongue muscle performance; and particularly on TE.

Findings of this study, however, revealed that weightlifters performed near the low end of healthy TE norms. Specifically, healthy adults are typically expected to be able to maintain a submaximal tongue muscle contraction for 25–35 sec; whereas a TE performance of 10 sec or less is considered less than optimal to maintain adequate tongue function (Crow and Ship 1996; Stierwalt and Youmans 2007; Adams et al. 2013). In the current study TE of weightlifters approached the low end of the previously published typical range; yet, none of the weightlifters reported any difficulty with functions that require tongue muscle endurance (i.e., swallowing, maintaining airway patency) during the prescreening process. It is important to mention that weightlifters tended to generate greater average tongue pressure (M* *=* *60.56 + SE* *= 2.62) than runners (M* *=* *54.24 + SE* *= 2.50). Consequently, weightlifters were also required to maintain a slightly higher tongue pressure than runners during the endurance task. In both groups, however, tongue pressure was within the normal range; therefore, it is unlikely that the difference in tongue pressure would explain the significant group effect for TE and the particularly low TE values for weightlifters.

### Group x tongue region interactions

As expected, TS was found to be significantly greater in the anterior region of the tongue than the posterior region of the tongue in weightlifters. Additonally, weightlifters produced significantly greater anterior TS than runners, whereas posterior TS was comparable between the two groups. Such findings are congruent with previous studies (Clark and Solomon 2012; Adams et al. 2013, 2014). First, the anterior tongue region is comprised predominantly of Type II fast‐twitch muscle fibers that are recruited in tasks requiring strength, speed, and power (Stal et al. 2003; Zaidi et al. 2013). Furthermore, weightlifting (i.e., resistance training) is known to yield a more pronounced effect on Type II fast‐twitch muscle fibers and muscular strength compared to endurance running (Holloszy and Coyle 1984; Wilson et al. 2012a).

Group x tongue region interaction were not significant for TE. Figure 4, however, suggests, that TE tended to be greater in the anterior tongue region than in the posterior region specifically for endurance runners. This finding concurs with previous studies reporting anterior TE to be greater than posterior TE (Adams et al. 2013, 2014). Within the context of previous studies on skeletal muscles (Karavirta et al. 2011; Wilson et al. 2012a,b), however, the observation of greater TE in the anterior tongue region than the posterior tongue region is rather incompatible with the current knowledge about muscle fibers that can be predominantly found within the two tongue regions (i.e., fatigue‐resistant type I and IM/IC fibers in the posterior region, fast fatiguing type II fibers in the anterior tongue region). Based on the region‐specific muscle fiber arrangements the posterior region of the tongue should yield greater TE values than the anterior region of the tongue. Hence, it is currently difficult to explain why particularly runners tended to demonstrate greater TE in the *anterior t*ongue region than in the posterior tongue region.

### Potential mechanisms underlying observed between‐group differences

Although the current study was able to uncover significant differences in tongue muscle performance between individuals who regularly engage in weightlifting and those who regularly engage in long‐distance running, the underlying mechanisms that may explain these differences remain unknown. In this brief section we speculate that between‐group differences in tongue muscle performance may be attributed to the specific breathing patterns that the two types of exercise are associated with. Specifically, weightlifters implement the Valsalva maneuver to increase rigidity and stability of the trunk. The forceful expiration against a closed glottis creates high subglottal pressures. To withstand such high subglottal pressures, supralaryngeal muscle groups assist with glottal closure. Furthermore, forceful inhalation and exhalation before and after the glottal closure require rapid changes in airway patency regulated by the posterior tongue region. Thus, regular engagement in a Valsalva maneuver may elicit muscular adaption of the tongue and increase strength.

In contrast to the breathing patterns of weightlifters, runners utilize a rhythmic breathing pattern that is synchronized with their limb movements. This form of breathing elicits muscle contractions at a submaximal level of effort and over an extended period of time with minimal fatigue 15, 17). The tongue muscle fibers may adapt to the increased demand of the submaximal contraction over an extended period of time to guarantee airway patency. Future studies are needed to investigate if and how tongue muscles are adapting to regular engagements in these two distinct breathing patterns to better understand the indirect effects of different modes of exercise on tongue muscle performance.

### Clinical implications

Previous studies have shown that tongue muscle performance (strength, endurance) declines with age and can negatively impact tongue function (Crow and Ship 1996; Nicosia et al. 2000; Clark and Solomon 2012; Adams et al. 2013). For example, presbyphagia is commonly associated with a significant age‐related decline in TS. Furthermore, a significant age‐related decline in both TS and endurance can be a contributing factor of developing an obstructed upper airway disorder known as sleep apnea (Blumen et al. 2004; McSharry et al. 2012).

The current study paves the way for future research investigating possible approaches to slow aging‐related decline on tongue muscle performance to prevent serious illness due to the decline of tongue muscle function. The findings of a recent study suggest that an active lifestyle (i.e., high level of physical activity) may reduce the aging‐related decline in TS and endurance (VanRavenhorst‐Bell et al. 2017). The current findings further suggest that the type of physical activity could differentially impact TS and endurance.

### Limitations and future directions

This study is the first effort to investigate *indirect* effects of physical exercise mode on tongue muscle performance in humans. Due to the relatively small sample size, findings should be interpreted with caution and replication studies are needed. Furthermore, the aims of this study were approached by examining between‐group differences in tongue muscle performance of individuals who regularly engage in either resistance training (weightlifting) or endurance training (running). Now that between‐group differences have been observed, pre/posttraining designs are warranted in future studies to examine the *indirect* effect of mode of exercise while controlling for other factors potentially impacting tongue muscle performance (e.g., genetic and anatomical factors that may steer individuals to choose a specific sport such as running or weightlifting).

Although the current study verified that each participant trained for 4 or more days per week for at least the past year within his or her specified mode of exercise (weightlifting, running) and controlled for cross‐training, other factors of exercise (e.g., *intensity, duration*) were not controlled. Studies have shown that such exercise factors can impact skeletal muscle strength and endurance (Karavirta et al. 2011); therefore these factors of exercise may have influenced the *indirect* effect a particular mode of exercise had on TS and endurance. Future studies should consider these factors to reduce within‐group variability.

## Conclusion

Findings of this study suggest that weightlifters produce significantly greater TS than runners, particularly in the anterior region of the tongue. By contrast, runners produced significantly greater TE than weightlifters. Future studies are needed to delineate the mechanisms underlying these between‐group differences. Such insight may yield important implications for the maintenance of healthy tongue muscle performance and prevention of aging‐related tongue muscle performance decline.

## Conflict of Interest

The authors report no conflicts of interest. The authors alone are responsible for the content and writing of the paper.
